# Editorial for the *IJMS* Special Issue on Sglt2 Inhibitors (Volume 2)

**DOI:** 10.3390/ijms242316865

**Published:** 2023-11-28

**Authors:** Anastasios Lymperopoulos

**Affiliations:** Laboratory for the Study of Neurohormonal Control of the Circulation, Department of Pharmaceutical Sciences, Nova Southeastern University Barry and Judy Silverman College of Pharmacy, Fort Lauderdale, FL 33328-2018, USA; al806@nova.edu; Tel.: +1-954-262-1338; Fax: +1-954-262-2278

The goal of the second volume of this Special Issue was to build upon the success of the first one and to continue to highlight the ever-expanding list of pharmacological properties of the sodium/glucose co-transporter (SGLT) type 2 (SGLT2) inhibitor (SGLT2i) drug class (also known as gliflozins). Originally developed for restoring euglycemia in those with diabetes mellitus, this drug class is now known to exert pleiotropic effects in the cardiovascular system independently of these drugs’ blood-glucose-regulating effects [1]. Indeed, thanks to these unrelated-to-diabetes effects, such as decreased mortality, amelioration of kidney function, increased hematocrit, lower blood pressure, weight loss, improved neurohormonal status of the myocardium, and direct induction of autophagy and improved mitochondrial energetics in the heart, most of these drugs are now considered a first-line treatment for heart failure regardless of diabetic status in the latest European Society of Cardiology (ESC)-issued guidelines [2]. Importantly, the various beneficial cardiovascular effects of these agents also appear to be unrelated to their main mechanism of action, i.e., SGLT2 inhibition. Most of their clinical effects (if not all), however, stem from the hypoglycemia they induce, which provokes an overall state of nutrient deprivation (fasting) in the body [1]. With these important notes in mind, this volume features some interesting studies (along with some important, comprehensive reviews) that explore the physiological and pharmacological effects of certain SGLT2is.

A few of them focused on empagliflozin, the most selective for SGLT2 among the currently available SGLT2is used in clinical practice [3,4]. Using an obese TallyHo mouse model, which recapitulates the human condition of diabetes and nonalcoholic fatty liver disease (NAFLD), Kurtz et al. reported that empagliflozin attenuated hepatic steatosis via expanding white adipose tissue (WAT) in this mouse model of obesity [5]. More specifically, empagliflozin decreased not only blood glucose levels, as expected, but also circulating triglyceride levels and lipid accumulation in the livers of male TallyHo mice. This correlated with lower levels of hepatic cholesterol esters [5]. Via in vivo MRI analysis, an empagliflozin-mediated reduction in hepatic steatosis in male TallyHo mice was found to be associated with an increase in nuchal white fat indicative of “healthy fat expansion”. Notably, this whitening of the fat cells occurred at the expense of brown adipose tissue (BAT) [5]. Although the precise molecular mechanisms underlying these effects of empagliflozin in adipose tissues await delineation in future studies, this study strongly suggests that SGLT2is (or at least empagliflozin) might be of use in treating non-alcoholic fatty liver disease (NAFLD) and in steatohepatitis pharmacotherapies. On the other hand, empagliflozin was found to reduce vascular calcification in mice with chronic kidney disease (CKD) by activating adenosine monophosphate (AMP)-activated protein kinase (AMPK), which regulates the nuclear factor erythroid 2-related factor (NRF)-2/heme oxygenase (HO)-1 anti-inflammatory signaling pathway [6]. This study used an inorganic-phosphate-induced vascular calcification model to investigate the mechanisms underlying empagliflozin’s therapeutic effects [6]. The authors evaluated biochemical parameters, such as mean artery pressure (MAP), pulse wave velocity (PWV), transcutaneous glomerular filtration rate (GFR), and histology, in an in vivo mouse model with vascular calcification induced via an orally administered high-phosphorus diet following a 5/6 nephrectomy. Empagliflozin lowered blood glucose, MAP, PWV, and vascular calcification in nephrectomized ApoE^−/−^ mice fed a high phosphorus diet, while it increased calcium levels and GFR [6]. It also inhibited osteogenic trans-differentiation by decreasing inflammatory cytokine expression and increasing AMPK, Nrf2, and HO-1 levels. In mouse vascular smooth muscle cells (VSMCs) in vitro, empagliflozin reduced high phosphate-induced calcification via activating AMPK/Nrf2/HO-1 signaling [6]. Thus, based on this study, empagliflozin can directly treat vascular calcification in animal models of this disease thanks to its well-documented and SGLT-independent effect on AMPK activation [7,8]. Interestingly, canagliflozin can also increase HO-1 levels and activity in human endothelial cells according to another study published in this volume, suggesting that HO-1 upregulation might be a property shared by the entire SGLT2i class.

Finally, the effects of empagliflozin in cardiac sympathetic activation and inflammatory cell infiltration due to angiotensin II (AngII)-dependent hypertension were tested by Castoldi et al. [9]. Empagliflozin administration prevented the development of myocardial hypertrophy, fibrosis, inflammatory cell infiltration, and tyrosine hydroxylase (the rate-limiting enzyme in catecholamine biosynthesis [10]) upregulation induced by Ang II treatment in Sprague-Dawley rats, indicating reverse-remodeling, anti-inflammatory, and sympatholytic effects for this SGLT2i [9]. Interestingly, empagliflozin did not affect blood glucose or Ang II-induced increase in blood pressure in these animals. These intriguing findings strongly indicate that empagliflozin’s cardioprotective effects in Ang II-dependent hypertension are not due to the direct suppression of an AngII-induced increase in peripheral vascular tone and blood pressure or to the lowering of blood glucose but instead might be due to the local suppression of cardiac inflammation and sympathetic hyperactivity in the context of AngII-mediated hypertension. Of note, another similar SGLT2 inhibitor, dapagliflozin, has been reported to exert sympatholysis with tyrosine hydroxylase downregulation and reductions in norepinephrine content in the heart as well [11,12,13]. Therefore, sympatholysis might be a key mechanism by which SGLT2is confer several of their beneficial effects on the diseased myocardium. More studies are needed to shed light on the exact mechanism(s) of SGLT2i-induced reductions in sympathetic nervous system activity and norepinephrine synthesis as well as to ascertain whether this is a class-wide effect or a property only certain agents within the class (e.g., dapagliflozin and empagliflozin) possess. Interestingly, a recent study documented a novel molecular target for dapagliflozin’s sympatholytic effects: the adrenal Regulator of G protein Signaling (RGS)-4 upregulation [14]. RGS4 belongs to the superfamily of GTPase-activating proteins (GAPs) for G proteins; i.e., it accelerates G_i/o_- and G_q/11_-protein-signaling termination [15]. RGS4 is abundantly expressed in the adrenal glands, both in the cortex and in the medulla [16], and its genetic deletion in mice is associated with elevated levels of circulating catecholamines, i.e., increased sympathetic activity, and elevated free fatty acid (FFA) levels in the blood [17]. Of note, one of the four G-protein-coupled receptors (GPCRs) mediating FFA signaling inside cells [18], FFA receptor type 3 (FFAR3), has been reported to promote norepinephrine release from sympathetic neurons [19], and FFAR3 signaling was recently shown to be inhibited by RGS4 in cardiomyocytes and sympathetic-like neurons [20]. In any case, adrenal RGS4 inhibits cholinergic (vagal)-nerve-stimulated catecholamine release from the chromaffin cells of the adrenal medulla [15,17], making it sympatholytic in this secretory organ. Consequently, dapagliflozin, by transcriptionally upregulating RGS4 in the adrenals [14], can have a direct sympatholytic effect via RGS4 in the adrenal medulla. Whether this property of dapagliflozin (i.e., RGS4 upregulation) is shared by other SGLT2is (i.e., making it a drug class-wide effect) or is unique to dapagliflozin remains to be determined.

Moreover, dapagliflozin exerts important antioxidant effects in lens epithelial cells (LECs) of animals fed a high-fructose diet to induce diabetes. SGLT2 levels, like those of glucose transporter (GLUT)-1, GLUT5, the reduced form of nicotinamide adenine dinucleotide phosphate (NADPH) oxidase (NOX) subunit p47/p67-phox, and NOX4, were significantly increased in LECs from diabetic animals compared to those from healthy controls. Importantly, dapagliflozin was able to reduce GLUT5, p47/p67-phox, NOX4, and the receptor for advanced glycation end products (RAGE) levels in diabetic LECs. Therefore, dapagliflozin can reduce oxidative stress and reactive oxygen species (ROS) production in diabetic ocular tissues; accordingly, SGLT2 inhibitors may have a place in the pharmacological treatment of cataracts and diabetic eye disease.

Finally, out of four SGLT2 inhibitors compared, only canagliflozin (but not dapagliflozin, ertugliflozin, or empagliflozin) improved mitochondrial energetics in human umbilical vein endothelial cells (HUVECs). Canagliflozin also inhibited GLUT1 (without affecting its expression levels), reduced glycolysis, increased levels of amino acids supplying the tricarboxylic acid cycle, and significantly increased levels of purine/pyrimidine pathway metabolites, whereas dapagliflozin and ertugliflozin had no effect. Thus, it seems that canagliflozin, but not dapagliflozin, ertugliflozin, or empagliflozin, inhibits mitochondrial activity and glucose uptake at both pharmacological and supra-pharmacological concentrations. The other three agents have no effect even at supra-pharmacological concentrations. If these findings hold true in vivo, then canagliflozin may be the least safe metabolically, given its strong inhibition of mitochondrial function and its suppression of cellular glucose uptake. Another intriguing question is why only canagliflozin would exert these effects but not any of the other SGLT2 inhibitors studied. Perhaps the answer lies in the fact that canagliflozin is the most potent inhibitor of SGLT1 among the four SGLT2is tested [3], so, if HUVECs express mainly SGLT1 rather than SGLT2, the reported results with canagliflozin would make sense. Obviously, more studies conducted on other types of endothelial cells and in vivo are warranted to confirm this intriguing finding.

In summary, the studies published in both the previous volume and this one clearly demonstrate the plethora and complexities of the mechanisms SGLT2 inhibitor drugs utilize, beyond the target they were originally developed to inhibit, i.e., SGLT2. They also highlight the multitude of cardiovascular effects they exert independently of their blood-glucose-lowering action. At the currently frenetic pace at which discoveries are made regarding novel actions and mechanisms for these medications, it will not be long before this remarkable drug class proves to be a game changer in even more medical disciplines and disease areas, having already conquered the fields of diabetes and heart failure pharmacotherapies.
